# Supplementary material for: An Analysis of Variability in the Content of Phenolic Acids and Flavonoids in Camelina Seeds Depending on Weather Conditions, Functional Form, and Genotypes
Source: Molecules. 2022 May 24;27(11):3364. doi: 10.3390/molecules27113364 (PMC9181862; doi:10.3390/molecules27113364)
Supplement: Supplementary file 1 [file molecules-27-03364-s001.zip › molecules-1725508-supplementary.pdf]

# Supplementary material

## An analysis of variability in the content of phenolic acids and flavonoids in camelina seeds depending on weather conditions, functional form, and genotypes

Danuta Kurasiak-Popowska[1], Małgorzata Graczyk[2], Anna Przybylska-Balcerek[3], Kinga Stuper-Szablewska[3]
Lidia Szwajkowska-Michałek[3]*

Table S1. List of spring and winter accessions of *C. sativa* used in this study

| | Accession | Plant name | origin |
|---|---|---|---|
| | | **Spring biotypes** | |
| 1 | | UP 2017/02 | Poland |
| 2 | Ames 31231 | GE2011-01 | Georgia |
| 3 | Ames 31232 | GE2011-05 | Georgia |
| 4 | PI 258366 | VNIIMK 17 | Former Soviet Union |
| 5 | PI 258367 | Voronezh349 | Former Soviet Union |
| 6 | PI 304268 | No. 401 | Sweden |
| 7 | PI 304269 | No.402 | Sweden |
| 8 | PI 304270 | No.403 | Sweden |
| 9 | PI 304271 | No. 406 | Sweden |
| 10 | PI 311735 | Borowska | Poland |
| 11 | PI 311736 | Przybrodzka | Poland |
| 12 | PI 597833 | 163-2073-72 | Denmark |
| 13 | PI 633192 | CR 476/65 | Germany |
| 14 | PI 633193 | CR 492/94a | Germany |
| 15 | PI 633194 | Giessen Nr. 3 | Germany |
| 16 | PI 650140 | Came | Germany |
| 17 | PI 650141 | NU 52279 | US, Minnesota |
| 18 | PI 650142 | CS -163-2073-72 | Denmark |
| 19 | PI 650143 | CS-CROO | Germany |
| 20 | PI 650145 | BRSCHW 28347 | Germany, Macklenburg-W.P. |
| 21 | PI 650146 | BRSCHW 30021 | Sweden |
| 22 | PI 650147 | Came | Sweden |
| 23 | PI 650148 | Giessen #3 | Germany, Macklenburg-W.P. |
| 24 | PI 650149 | Giessen #4 | Germany, Macklenburg-W.P. |
| 25 | PI 650150 | Hoga | Denmark |
| 26 | PI 650151 | Svalof | Sweden |
| 27 | PI 650152 | CPS-CAM23 | Germany |
| 28 | PI 650153 | CPS-CAM10 | Former Soviet Union |
| 29 | PI 650154 | CSS-CAM25 | Former Soviet Union |
| 30 | PI 650155 | CSS-CAM27 | Poland |

| | | | |
|---|---|---|---|
| 31 | PI 650156 | CSS-CAM29 | Former Soviet Union |
| 32 | PI 650157 | CSS-CAM30 | Former Soviet Union |
| 33 | PI 650158 | CSS-CAM31 | Poland |
| 34 | PI 650159 | CSS-CAM33 | Poland |
| 35 | PI 650160 | CSS-CAM34 | Former Soviet Union |
| 36 | PI 650161 | CSS- CAM35 | Former Soviet Union |
| 37 | PI 650162 | CSS-CAM36 | Poland |
| 38 | PI 650163 | CSS-CAM37 | Former Soviet Union |
| 39 | PI 650164 | CSS-CAM38 | Austria |
| 40 | PI 650165 | CSS-CAM7 | Former Soviet Union |
| 41 | PI 650166 | CSS-CAM8 | Former Soviet Union |
| 42 | PI 650167 | Index Seminum144 | Poland |
| 43 | PI 650168 | NE2006-1 | US, Nebraska |
| 44 | PI 652885 | 1 | Slovenia |
| 45 | PI 652886 | 4 | Slovenia |
| 46 | | 1025 | Poland |
| 47 | | 515 | Poland |
| 48 | | 516 | Poland |
| 49 | | 514 | Poland |
| 50 | | 7 | Poland |
| 51 | | 8III/2. | Poland |
| 52 | | 44 | Poland |
| 53 | | 2 | Poland |
| 54 | | L-3 | Poland |
| 55 | | Omega | Poland |
| 56 | | Zarja Socializma | Ukraine |
| 57 | | Zavolzskij | Ukraine |
| 58 | | GZPK | Switzerland |
| 59 | | Kirgzkij | Ukraine |
| 60 | | Sortandinskij | Ukraine |
| 61 | | Urkajinskij | Ukraine |
| 62 | | Omskij Mestnyj | Ukraine |
| 63 | | Grodziska | Ukraine |
| **Winter biotypes** | | | |
| 64 | | Przybrodzka | Poland |
| 65 | | 14 2 2 | Poland |
| 66 | | Luna | Poland |
| 67 | | Maczuga | Poland |
| 68 | | C5 | Poland |
| 69 | | Lenka | Poland |
| 70 | | K 9/1 | Poland |
| 71 | | 14/2/3 | Poland |
| 72 | | K 10/1 | Poland |
| 73 | | 15/2/2 | Poland |
| 74 | | 15/2/3 | Poland |

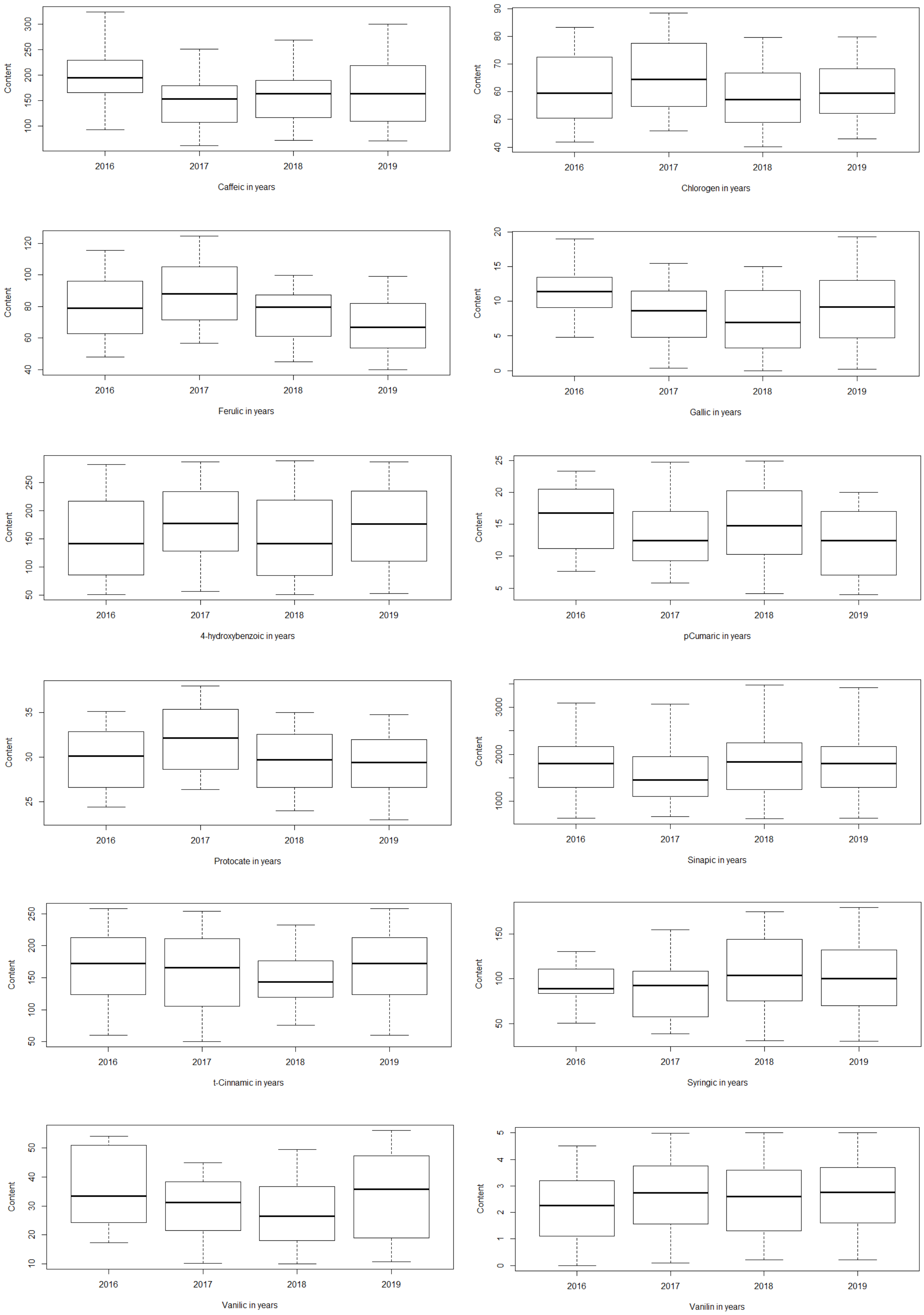


Figure S1. The boxplots for polyfenols and varieties

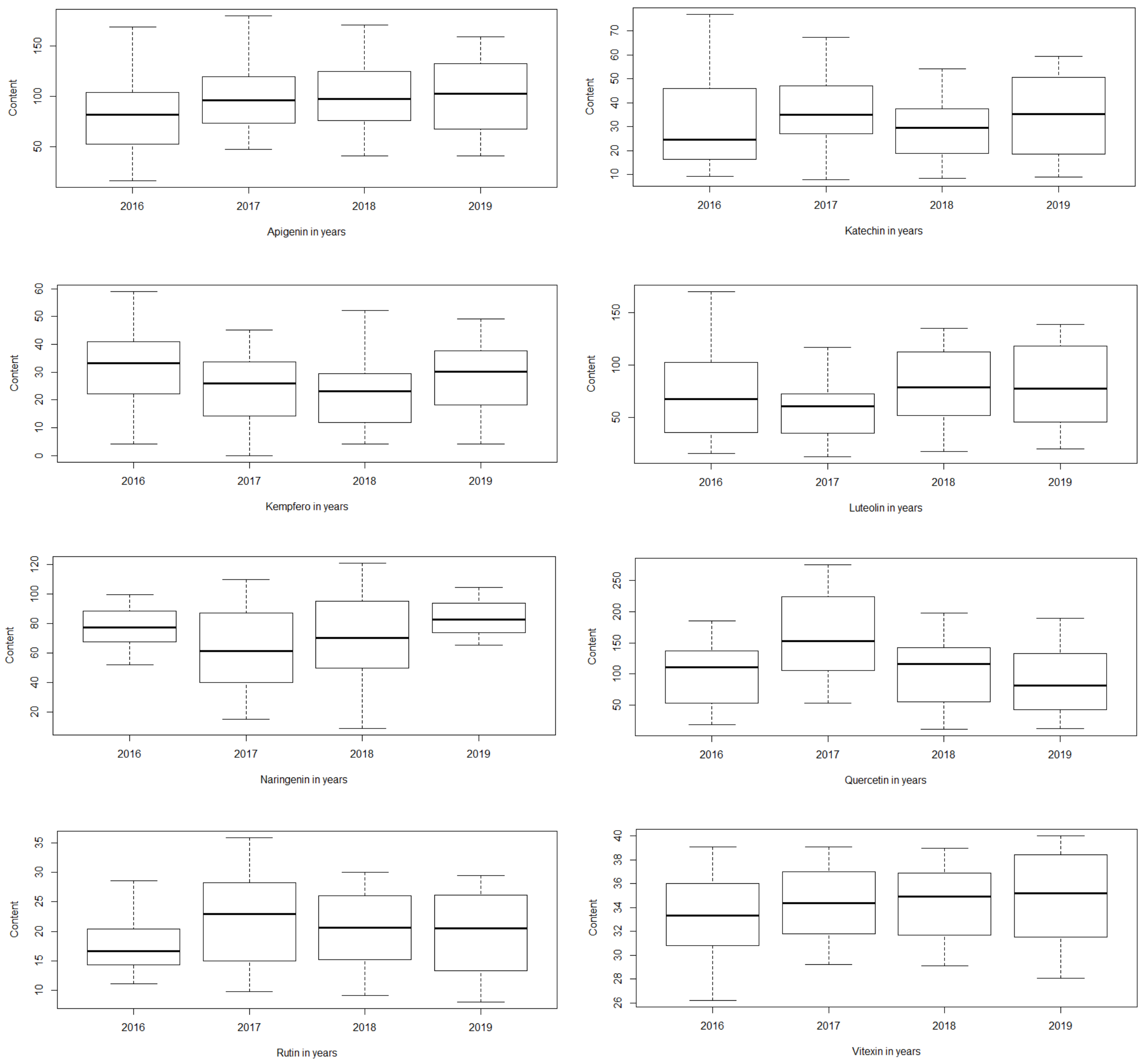


Figure S2. The boxplots for flavonoids and varieties
